# An Internally Controlled Quantitative Target Occupancy Assay for Covalent Inhibitors

**DOI:** 10.1038/s41598-018-32683-w

**Published:** 2018-09-25

**Authors:** Rasmus Hansen, Sarah J. Firdaus, Shuangwei Li, Matthew R. Janes, Jingchuan Zhang, Yi Liu, Patrick P. Zarrinkar

**Affiliations:** 1 Wellspring Biosciences, Inc., 3033 Science Park Rd., Ste. 220, San Diego, CA 92121 USA; 2 Kura Oncology, Inc., 3033 Science Park Rd., Ste. 220, San Diego, CA 92121 USA

## Abstract

Assessing target occupancy is critical for establishing proof-of-mechanism for novel inhibitors and to determine whether robust target inhibition can be achieved at tolerated doses. This is challenging in the clinic using conventional methods due to the need for untreated controls. We describe a new mass spectrometry approach to quantitatively assess target occupancy for covalent inhibitors that does not require untreated controls, and apply the method to the KRAS^G12C^ inhibitor ARS-1620.

## Introduction

An important challenge in translational research is to accurately and quantitatively assess target occupancy by inhibitors in preclinical and clinical samples. Understanding the relationship between target occupancy and biological effect is critical for determining whether adequate target inhibition can be achieved at tolerated exposures, what extent of target engagement is required to elicit the desired biological effect, and for establishing a mechanistic link between efficacy and target inhibition^1–3^. Target occupancy is typically assessed by measuring biomarkers that represent a direct readout of target activity, such as the phosphorylation state of the substrate of a kinase target. For covalent inhibitors, alternative approaches have been described that take advantage of the stable bond between inhibitor and target. These include assays that use labeled probe compounds to quantitate the remaining free, unoccupied target in specimens taken from animals or patients pre- and post-treatment with the test compound^4,5^, or direct quantitation by mass spectrometry of the remaining free target not covalently bound to inhibitor^6^. For all of these approaches, the absolute level of unoccupied target or phosphorylated substrate is measured in treated samples, and percent occupancy is determined by comparing this signal to the signal in untreated or pre-treatment controls. The requirement for such controls represents a major limitation, particularly during clinical development for solid tumors. For hematologic malignancies, it is possible to assess target occupancy in tumor cells using blood samples, which in the context of clinical development can be obtained relatively frequently from patients, including pre- and post-treatment samples taken only hours apart. For solid tumors, however, target occupancy assays in tumor tissue require material from tumor biopsies. Solid tumor biopsies typically cannot be performed in a serial fashion in the clinic, nor can matched biopsies from untreated patients be readily obtained. New methods for the accurate determination of target occupancy in solid tumors that do not require pre-treatment or untreated controls therefore represent a significant unmet need.

The clinical success of covalent kinase inhibitors^7,8^, and the discovery that KRAS, until recently considered undruggable, can be targeted with covalent inhibitors that react with the mutated cysteine in KRAS^G12C^, one of the most common oncogenic mutant variants of KRAS^6,9–13^, has generated renewed interest in covalent inhibitors as a class^14^. To facilitate the development and clinical assessment of covalent inhibitors, we now describe a novel approach for quantitating target occupancy that does not require comparison to untreated or pre-treatment controls and directly yields percent occupancy values from individual biological samples. The method uses internal standards added to samples during processing to allow absolute quantitation of both covalently bound and unbound target by mass spectrometry. We apply the method to ARS-1620, the first covalent KRAS^G12C^ inhibitor shown to engage and inhibit KRAS *in vivo*, inducing tumor regressions in a wide range of KRAS^G12C^ cell and patient derived xenograft models^6^.

## Results

### Quantitating free and bound target protein

To address the need for determining the percent target occupancy for covalent inhibitors from individual samples, we decided to independently quantitate by mass spectrometry the absolute amounts of both free and covalently bound target protein. Quantitating both free and bound target allows the internal calculation of percent occupancy from free or bound relative to total target within each individual sample, without requiring any untreated controls. This strategy therefore overcomes a major limitation of current approaches. As a proof-of-concept case we focused on the recently described covalent KRAS^G12C^ inhibitor ARS-1620^6^. ARS-1620 covalently reacts with the mutant cysteine 12 of KRAS^G12C^ in the signaling-incompetent KRAS^G12C^-GDP complex, thereby inhibiting nucleotide exchange and thus formation of the signaling-competent KRAS^G12C^-GTP complex.

**Figure 1:**
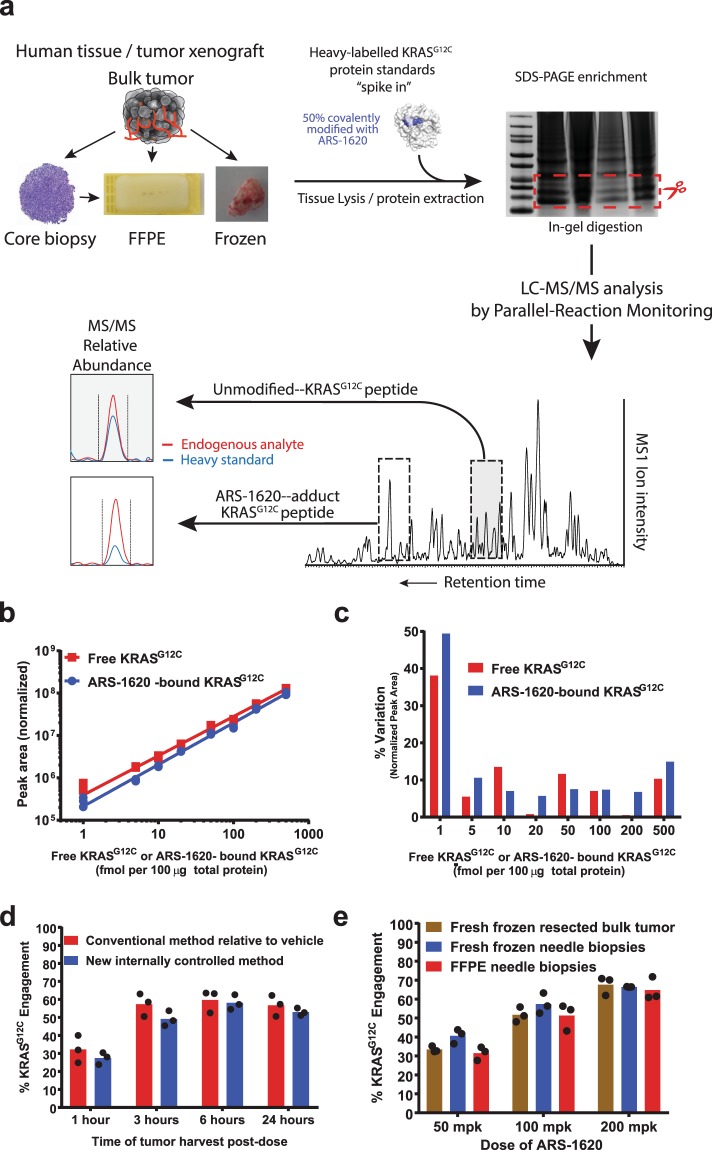
Validation of the ARS-1620 KRAS^G12C^ occupancy assay. (**a**) Assay workflow. An uncropped version of the gel image is shown in the accompanying Supplementary Information. (**b**,**c**) Determination of l.o.d. and l.o.q. of free and ARS-1620-bound KRAS^G12C^ protein. (**b**) Sensitivity of detection. Individual replicates are shown. For most, the points closely overlap. A fit of the data to straight lines yielded R^2^ values of 0.99 for both free and ARS-1620-bound KRAS^G12C^. (**c**) Percent Variation calculated as the percent of the mean of the range of values observed [100 × ((max value − min value)/mean)] at different amounts of total spiked KRAS^G12C^ protein, calculated from the replicates shown in (**b**). (**d**) Comparison of the new internally controlled method to the conventional method of normalizing to vehicle treated controls. (**e**) Comparison of engagement values obtained from fresh-frozen resected bulk tumors, fresh-frozen needle biopsies, and FFPE needle biopsies, prepared from the same mouse xenograft tumors. In (**d**) and (**e**) bars indicate means calculated from the individual replicate values shown shown as black circles.

To enable quantitation of endogenous free and ARS-1620-bound KRAS^G12C^, sample lysates were spiked with a known amount of recombinant, isotopically mass-labeled free and inhibitor-bound KRAS^G12C^ as an internal standard (typically a 1:1 mixture of free and inhibitor-bound). KRAS^G12C^, including the endogenous protein as well as the spiked standard, was enriched from lysates by gel electrophoresis and protease digested to generate a specific peptide spanning the site of covalent modification from both inhibitor bound and unbound protein. Protease digested samples were analyzed by LC/MS-MS mass spectrometry to quantitate the endogenous free and inhibitor-bound peptide peaks along with the peaks for the internal standard, differentiated from the endogenous peaks by their mass label (Fig. 1a). For KRAS, trypsin digestion generates the peptide LVGA**C/G**GVGK containing residue 12. Residue 12 is cysteine in KRAS^G12C^, the target of covalent modification by ARS-1620, and glycine in wild type KRAS, which cannot be modified by ARS-1620. Due to the difference in molecular mass created by the oncogenic glycine to cysteine mutation, the free and ARS-1620-bound KRAS^G12C^-derived peptides can be individually and specifically detected and quantitated by mass spectrometry in the presence of wild type KRAS. The absolute amounts of free and ARS-1620-bound KRAS^G12C^ in each sample are determined by multiplying the ratio of the respective endogenous peptide peak abundance relative to the internal standard peptide peak abundance by the known amount of internal standard that had been spiked into the sample lysate (typically 0.5 fmol each of free and ARS-1620-bound isotopically labeled KRAS^G12C^ per microgram of lysate, see Methods). The engagement or occupancy fraction may then be calculated according to the following formula:

$${\rm{ \% }}{\rm{K}}{\rm{R}}{\rm{A}}{\rm{S}}{\rm{G}}12{\rm{C}}\,{\rm{e}}{\rm{n}}{\rm{g}}{\rm{a}}{\rm{g}}{\rm{e}}{\rm{m}}{\rm{e}}{\rm{n}}{\rm{t}}=\,\frac{ARS1620\,bound\,KRA{S}^{G12C}}{Free\,KRA{S}^{G12C}+ARS1620\,bound\,KRA{S}^{G12C}}$$

### Determination of sensitivity, precision, accuracy and reproducibility

To determine the sensitivity and linear range of detection of free and ARS-1620-bound KRAS^G12C^, we spiked decreasing amounts of recombinant free or ARS-1620-reacted KRAS^G12C^ protein, along with a constant amount of ^13^C^15^N-arginine/^13^C^15^N-lysine isotopically labeled internal standard, into lysates from mouse xenograft tumors generated from the human A375 melanoma cell line, which does not harbor a KRAS^G12C^ allele (see Methods for details on preparation of the internal standard). The spiked lysates were enriched for KRAS by gel electrophoresis, trypsin digested and processed as described in Methods, and the free and ARS-1620-bound LVGACGVGK peptides quantitated by mass spectrometry (Fig. 1b). Detection of free and bound peptides was linear from 500 to 5 fmol protein input per 100 µg lysate, with a percent variation [calculated as the percent of the mean of the range of replicate values observed, 100 * ((max value − min value)/mean)] from three replicate samples below 15% from 500 to 5 fmol. At 1 fmol/100 µg lysate the percent variation increased to 35–50% (Fig. 1c). Similar sensitivity was observed when recombinant free and ARS-1620-bound KRAS^G12C^ protein was spiked into lysate from a resected human tumor sample that did not harbor a KRAS mutation (Supplementary Fig. S1). Based on these data we designated the limit of detection (l.o.d.) of the assay as 1 fmol, and the limit of quantitation (l.o.q.) as 5 fmol for both free and ARS-1620-bound protein in 100 µg lysate, with a linear range to at least 500 fmol per 100 µg lysate.

To determine the technical precision and accuracy across a range of percent engagement values, we varied the ratio of ARS-1620-bound and free KRAS^G12C^ from 10 to 90% covalent adduct relative to free protein, keeping the total protein amount constant at 100 fmol per 100 µg lysate, and generated 3 independent samples for each ratio. The measured average percent occupancies for the three replicate samples at each ratio were within 8 percentage points of the true value, and the percent variation for the replicate samples at each adduct percentage was no more than 12 percent (Supplementary Table S1). Similar results were obtained when recombinant KRAS^G12C^ protein was spiked into a KRAS^G12C^-negative human tumor lysate (Supplementary Table S2).

To compare the performance of the new approach, where both free and ARS-1620-bound protein are quantitated in each sample, to a more traditional approach where only free protein is quantitated and percent occupancy determined by comparing treated samples to untreated controls^6^, we analyzed tumor samples from a mouse xenograft study using the KRAS^G12C^-positive human pancreatic cancer cell line MIA PaCa-2 both ways. In this study, mice bearing subcutaneous MIA Paca-2 tumors were treated with a single dose of ARS-1620 at 200 mg/kg or with vehicle control, and tumors harvested 1, 3, 6 and 24 hours after dosing, with three mice per time point in each cohort. The results were very consistent across the two methods (Fig. 1d), further validating the new approach.

To determine the reproducibility of the assay, we divided the fresh lysates from two of the tumors from the 1 hour cohort and two from the 6 hour cohort in the above study into four aliquots, which were frozen independently. Once a week over four weeks one of the lysate aliquots from each tumor was processed and target engagement assessed using the new method. The absolute variation across the four aliquots from each tumor was comparable for both 1 hour and 6 hour samples, with 6 to 12 percentage points variation for the 1 hour samples and 9 to 10 percentage points for the 6 hour samples. The variation relative to the mean value, however, was greater for the 1 hour samples than for the 6 hour samples due to the smaller mean values, and ranged from 14 to 42% for the four weekly replicates for the four tumors (Supplementary Table S3). The relative technical variability therefore is greater when the percent engagement is lower.

### Application of the assay to tumor biopsy specimens

Obtaining fresh frozen biopsies from patients in the context of clinical trials is logistically extremely challenging and expensive. Standard clinical practice is to prepare formalin fixed paraffin embedded (FFPE) blocks from tumor biopsies. We therefore tested whether FFPE samples would perform similar to fresh frozen tissue from the same tumor, using mouse xenograft tumors as test material. Mice bearing subcutaneous tumors from either the human non-small cell lung cancer cell line H1373 or from the MIA Paca-2 cell line, both harboring a KRAS^G12C^ mutation, were dosed orally with ARS-1620 at 50, 100 and 200 mg/kg, and tumor samples collected 4 hours after a single dose. Each tumor was resected and processed in three ways. One, a section of the resected tumor was processed as bulk tissue and immediately frozen in liquid nitrogen. Two, a needle biopsy was performed on the tumor and the biopsied tissue immediately frozen in liquid nitrogen. Three, a second needle biopsy was performed and the biopsied tissue added to formalin fixing fluid and paraffin embedded. FFPE samples were treated with a protocol specifically optimized for this purpose as described in Methods. The three sample types yielded comparable results, with the extent of engagement increasing with the dose of ARS-1620 as expected (Fig. 1e).

To test whether the KRAS^G12C^ engagement assay is sufficiently sensitive to determine target occupancy in human clinical samples, we obtained twelve FFPE specimens from human non-small cell lung tumors, including curls taken from FFPE blocks prepared from tumor resections and core needle biopsy FFPE samples (Table 1). Eleven of the samples were from KRAS^G12C^-positive tumors, one was from a patient with a KRAS^G12D^ mutation and served as a negative control. While the patients had not been treated with ARS-1620, our aim was to determine whether the KRAS^G12C^ LVGACGVGK peptide could be detected at a level above the l.o.q. determined above. The KRAS^G12C^ peptide could be detected above the l.o.q. in nine out of the eleven G12C-positive samples, in amounts corresponding to 11 fmol to 68 fmol KRAS^G12C^ protein per 100 µg lysate, and was not detected in the G12D mutant sample (Table 1). To quantitatively determine the percent target occupancy requires that both the free and bound protein are detected above the limit of quantitation. The lowest and highest levels of target engagement that can be quantitatively determined in tumors treated with inhibitor will therefore depend on the absolute combined amount of free and bound protein detected relative to the l.o.q. of 5 fmol per 100 µg lysate, as follows:

$$\begin{array}{rcl}{\rm{Fraction}}\,{\rm{Engaged}}\,(\mathrm{low}) & = & 5/({\rm{fmol}}\,{\rm{free}}\,{\rm{target}}\,{\rm{protein}}\\  &  & +\,{\rm{fmol}}\,{\rm{bound}}\,{\rm{target}}\,{\rm{protein}})\\ {\rm{Fraction}}\,{\rm{Engaged}}\,(\mathrm{high}) & = & 1-[5/({\rm{fmol}}\,{\rm{free}}\,{\rm{target}}\,{\rm{protein}}\\  &  & +\,{\rm{fmol}}\,{\rm{bound}}\,{\rm{target}}\,{\rm{protein}})]\end{array}$$

**Table 1:** Quantitation of KRAS^G12C^ mutant protein in human clinical FFPE tumor specimens.

Sample type	Sample Number	KRAS variant	Tumor Type	Tumor content^a^	Total protein recovery (μg)	KRAS^G12C^ protein (fmol per 100 μg)	Range of engagement values within quantifiable limits^b^
FFPE resected tumors	1	G12C	Adenocarcinoma	100%	180	48	10–90%
	2	G12C	Adenocarcinoma	100%	95	34^c^	15–85%
	3	G12C	Adenocarcinoma	90%	120	3	Outside quantifiable range
	4	G12C	Adenocarcinoma	25%	88	19^c^	26–74%
	5	G12C	Adenocarcinoma	65%	71	34^c^	15–85%
	6	G12C	Adenocarcinoma	60%	36	19^c^	26–74%
	7	G12C	Squamous cell carcinoma	60%	160	11	45–55%
	8	G12C	Squamous cell carcinoma	50%	120	17	29–71%
	9	G12D	Adenocarcinoma	70%	100	0	No signal
FFPE core tumor biopsies	10	G12C	Squamous cell carcinoma	40%	640	3	Outside quantifiable range
	11	G12C	Adenocarcinoma	65%	330	68	7–93%
	12	G12C	Adenocarcinoma	30%	200	20	25–75%

^a^Tumor content as specified by the supplier, based on hematoxylin and eosin (H&E) staining. ^b^Values are based on the number fmol KRAS^G12C^ protein detected per 100 μg total protein, as shown in the previous column in this table, and the calculation of the high and low limit of engagement as outlined in the text. Engagement values outside of the quantifiable limits may still be assigned as being above or below the quantifiable limits, as discussed in the text. ^c^For samples with less than 100 µg total protein recovered, the value shown represents the amount of KRAS^G12C^ detected in the entirety of the sample.

For example, if there were 5 fmol bound protein and 45 fmol free, both would be within the quantifiable range and the percent target occupancy could be accurately determined as 5 fmol/(5 fmol + 45 fmol), or 10%. However, if the amount of bound protein were lower than 5 fmol, and thus not quantifiable, while 50 fmol free protein was detected, an accurate percent occupancy could not be calculated, yet it would be possible to state that the occupancy is below 10%. The sensitivity of quantitating percent target engagement therefore depends on the total amount of target protein detected (Table 1). Together, these data show that KRAS^G12C^ target engagement for the covalent inhibitor ARS-1620 can be quantitatively determined from FFPE samples, including clinical tumor specimens, without the need for pre-treatment or untreated control samples.

## Discussion

The quantitative determination of target occupancy is a significant challenge in translational research. For covalent inhibitors, current methods directly or indirectly quantitate free target remaining in a given sample, and rely on comparison to untreated controls or samples obtained prior to treatment to infer the percent target coverage. This requirement for control samples represents a major limitation, particularly for development of agents for the treatment of solid tumors. While serial blood samples, including pretreatment samples, may be obtained from patients, or indeed from animals in preclinical studies, and thus may be used to assess target occupancy for leukemia, solid tumor biopsies for most tumors typically cannot be performed in a serial fashion. Patient-matched reference biopsies are not typically available during clinical development either, making it difficult or impossible to quantitate target occupancy accurately for covalent agents. The approach we describe here, to independently quantitate both free and inhibitor-bound target within individual samples, provides a potential solution to this challenge. Quantitation of free and inhibitor-bound target is enabled by spiking each sample with a known amount of an internal standard consisting of an equal mixture of mass-labeled free and bound target protein identical to the endogenous target except for the mass label. Both free and bound target may then be quantitated by comparison to the respective standard. While the use of mass-labeled internal standards is common practice in mass spectrometry, the application of a mixture of free and inhibitor-bound standards to solve the specific problem of quantitating target occupancy for covalent inhibitors in tumor specimens, without the requirement of untreated control samples, has not been previously described and fills a significant unmet need.

The strategy described here should be readily applicable to other covalent inhibitors and other targets. The only requirement is the availability of mass-labeled free and inhibitor-bound target protein to create the internal standard. Any protein that can be produced with recombinant technology may be generated in isotopically labeled form as described here. The inhibitor-bound form may then be generated by reacting the mass-labeled target protein with the test compound in a biochemical reaction. This may be readily achieved as long as the mass-labeled protein can be produced in a purified form that may be targeted and covalently modified by the inhibitor. One potential limitation of the approach is that the target protein must be present at levels above the limit of quantitation by mass spectrometry. Proteins expressed at very low levels, or for which the peptide containing the targeted cysteine has low mass spectrometry ionization efficiency and ion transmission, may therefore prove challenging.

The approach described here represents a fit-for-clinical as well as preclinical use pharmacodynamics assay that may be applied to assess target occupancy by covalent inhibitors in the context of preclinical studies as well as clinical trials. We have applied the approach to the KRAS^G12C^ inhibitor ARS-1620, and demonstrate that the new strategy provides a more quantitative option for measuring target occupancy that does not require vehicle treated or pretreatment controls.

## Methods

### Inhibitor

ARS-1620 was synthesized as described^6^.

### Recombinant proteins

Recombinant KRAS^G12C^ protein (aa 1–169, histidine tagged) was produced as described^11,13^. Isotopically labelled KRAS^G12C^ (^13^C^15^N-Lys and ^13^C^15^N-Arg) was produced by PROMISE Advanced Proteomics (Grenoble, France) and modified with ARS-1620 using previously published biochemical reaction conditions^13^. The prepared internal standard was a molar 1:1 mixture of free and ARS-1620-reacted protein, as determined by LC-MS mass spectrometry.

### Clinical specimens

Clinical FFPE specimens were purchased from Conversant Bio (Huntsville, AL) and Asterand Bioscience (Detroit, MI). Conversant and Asterand comply with all applicable regulations.

### Animal studies

Mice were maintained under specific pathogen-free conditions, and food and water were provided *ad libitum*. All mouse experiments were approved by a local Animal Care and Use Committee and studies were conducted at an AAALA-accredited institution (Explora BioLabs; San Diego, CA) in accordance with all relevant guidelines and regulations.

### Sample preparation from frozen xenograft tumor specimens

Cell line derived tumor xenograft models were established in 6 to 8 week old female athymic BALB/c nude (NCr) nu/nu mice (Simonsen Labs; Gilroy, CA). MIA PaCa-2, H1373 or A375 cells (5 × 10^6^) were implanted subcutaneously in one flank in growth factor reduced Matrigel^TM^/PBS (50% Matrigel^TM^ in 100 µL injection volume). When tumors reached an average size of 200–400 mm^3^, mice were randomized into treatment groups as specified for each study. Vehicle (100% Labrasol) or compound were administered by oral gavage (10 mL/kg). At the indicated times, mice were sacrificed, tumors were harvested and processed as indicated. For fresh-frozen samples, specimens were snap frozen in liquid nitrogen and stored at −80 °C. To mimic biopsies from human patients, specimens were prepared from freshly harvested tumors using an 18-gauge punch needle and snap frozen. Frozen resected or needle biopsy tumor samples were homogenized using a Precellys 24 homogenizer (Bertin Instruments; Montigny-le-Bretonneux, France) using 2.0 mm Zirconia Beads (BioSpec Products; Bartlesville, OK) and solubilized in ice-cold lysis buffer (Pierce™ IP Lysis Buffer; Thermo Fisher Scientific) supplemented with cOmplete EDTA-free protease inhibitor cocktail Tablet (MilliporeSigma). Tumor lysates were centrifuged for 5 minutes at 17,000 x g to remove insoluble debris, supernatant recovered and protein concentration measured using the Pierce™ BCA Protein Assay Kit (Thermo Fisher Scientific). An aliquot of 100 to 400 μg total protein from each tumor lysate was spiked with 1 fmol isotopically labeled his-tagged recombinant KRAS^G12C^ 1–169 internal standard (prepared as described above as a 1:1 mixture of free and ARS-1620-bound protein) per μg total protein. Typically 100 μg of tumor lysate were used, and therefore spiked with 100 × 1 fmol = 100 fmol of internal standard. Where indicated, samples were also spiked with unlabeled recombinant KRAS^G12C^ and ARS-1620-reacted KRAS^G12C^ at different ratios in non-G12C tumor background matrices to evaluate assay performance. Following spiking of lysates with protein standards, total protein was precipitated by incubating samples with 1 mL acetone at −20 °C for a minimum of two hours, before proceeding with SDS-PAGE and in-gel digestion.

### Sample preparation from Formalin-Fixed Paraffin-Embedded (FFPE) tumor specimens

Cell line derived xenograft tumors were grown and harvested as described above. To generate FFPE specimens, bulk freshly harvested tumor or material extracted with an 18-gauge punch needle were treated with formaldehyde and paraffin embedded using a Leica Tissue Embedder. For resection samples, a 40 μm slice was cut from the block of paraffin-embedded bulk tumor and placed in a 1.5 mL microcentrifuge tube. For needle biopsy samples, the entire paraffin block containing the tumor biopsy was used. The paraffin wax around the tumor biopsy was first shaved off and the embedded tumor piece then placed into a 1.5 ml microcentrifuge tube. To remove the paraffin, 1 ml xylene was added and samples were vortexed and heated for 15 minutes at 56 °C. After removing liquid, the xylene extraction step was repeated twice more. To remove traces of formaldehyde and xylene, 100% ethanol was added to the tumor pieces or biopsy and left for 10 minutes at room temperature before removing the ethanol. The 100% ethanol wash step was repeated for a total of three times. The wash step was then repeated three times with 96% ethanol, and a further three times with 70% ethanol, again leaving the sample for 10 minutes at room temperature each time. The washed tumor samples were homogenized in 100 µl buffer (20 mM Tris pH 8.0, 1% SDS, 5% glycerol) using a motorized pestle, followed by sonication using a Sonifier® Cell Disruptor Model SLPe (Branson Ultrasonics; Danbury, CT). The homogenized samples were incubated in a heating block at 90 °C for 90 minutes. Following homogenization, tumor lysates were centrifuged for 5 minutes at 17,000 x g to remove insoluble debris, supernatant recovered and protein concentration measured using the Pierce™ BCA Protein Assay Kit (Thermo Fisher Scientific). The supernatant lysates were spiked with 1 fmol of a 1:1 mixture of free and ARS-1620-modified isotopically labelled his-tagged recombinant KRAS^G12C^ 1–169 internal standard per µg total protein, as described above. Following spiking of the lysates with internal standard, total protein was precipitated by adding 1 mL acetone and incubating at −20 °C for a minimum of two hours before proceeding with SDS-PAGE and in-gel digestion.

### In-gel protease digestion

After acetone precipitation, lysates were centrifuged at 13,000 x g for 5 minutes, and the supernatant removed. Protein pellets were resuspended in 1X LDS sample buffer containing 50 mM dithiothreitol, incubated at 70 °C for 10 minutes, and separated by SDS-PAGE (8% Bis-Tris gels, Invitrogen) alongside size standards (Precision Plus Protein Dual color; BioRad; Hercules, CA). Separated proteins were visualized with Coomassie staining (SimplyBlue SafeStain; Invitrogen). A ChemiDoc MP imaging system (BioRad; Hercules, CA) was used to image protein bands, using Image Lab v4.1 software and white trans illumination with a standard filter to obtain the gel image. A gel piece corresponding to 15 to 25 kDa based on the size standards was excised from each gel lane, cut into approximate 1 mm^3^ cubes and placed in a microcentrifuge tube. Five hundred µL of 100% acetonitrile was added to the gel pieces and incubated for 5 minutes at room temperature to dehydrate the gel pieces. After removing the acetonitrile, 500 µL of 10 mM dithiothreitol in 50 mM ammonium bicarbonate was added to reduce disulfide bonds, and samples were incubated for 30 minutes at 56 °C. The dithiothreitol solution was removed and the gel pieces washed in 1 mL 30% acetonitrile/50 mM ammonium bicarbonate for 30 minutes at room temperature. Five hundred µL of 50 mM iodoacetamide in 50 mM ammonium bicarbonate was added to alkylate free cysteines, and samples were incubated for 30 minutes at 37 °C. The iodoacetamide solution was removed and the gel pieces washed in 1 mL 30% acetonitrile/50 mM ammonium bicarbonate for 30 minutes at room temperature. Five hundred µL of 100% acetonitrile was added to the gel pieces and samples incubated for 5 minutes at room temperature to dehydrate the gel pieces. After removing the acetonitrile, proteins were digested in 150 μl of 20 ng/μl sequencing-grade trypsin (Promega; Madison, WI) in 5% acetonitrile/20 mM ammonium bicarbonate overnight at 37 °C. The tryptic digest produces the KRAS peptide spanning residue 12, used for quantitating free and ARS-1620-bound KRAS^G12C^ (Supplementary Table S4). The supernatant from the digestion reaction was recovered and peptides were extracted from the gel pieces with 100 µL of 30% acetonitrile for 30 minutes at room temperature. The supernatant was combined with the supernatant from the trypsin digest reaction. Lastly, 100 µL of 100% acetonitrile was added to the gel pieces and incubated for 5 minutes at room temperature to further extract any remaining peptides. The supernatant was combined with the previous digest and extraction supernatants and samples were dried by evaporation in a vacuum centrifuge. The samples were stored at −20 °C until LC-MS/MS analysis.

### Mass spectrometry, LC-MS/MS

Peptides from the trypsin digests were resuspended in 6 μl 50% acetonitrile, and acidified with 54 μl 0.2% formic acid. A volume of 10 μl was analyzed by LC-MS/MS. Peptides were separated on a 15 cm C18 reverse-phase analytical column with integrated heater and emitter (Thermo EasySpray) at a flow rate of 600 nl/min using a Dionex RSLCnano LC (Thermo Fisher Scientific). Peptides were eluted over a 15 minute gradient ranging from 6 to 30% acetonitrile/0.1% formic acid. A Q Exactive™ quadrupole orbitrap mass spectrometer (Thermo Fisher Scientific) was run alternating between full MS acquisition and time-scheduled targeted MS2 mode, also known as Parallel Reaction Monitoring (PRM) mode. The sequences and inclusion list information for targeted precursor peptide ions in MS2 mode are listed in Supplementary Table S4. Full MS spectra were collected at a resolution of 17,500, with an AGC target of 1 × 10^6^ or maximum injection time of 50 ms and a scan range of 380–1750 m/z. The MS2 spectra were obtained at a resolution of 37,500, with an AGC target value of 3 × 10^6^ or a maximum ion injection time of 120 ms at a quadrupole isolation width of 1.6 m/z. Normalized collision energy was set to 27 au for all peptides. The raw data were processed using Skyline version 3.6 (University of Washington; Seattle, WA). The peak area for each peptide precursor was the sum of the total integrated area for the two selected fragment ions (Supplementary Table S4) within automatically set chromatographic peak boundaries. The signal for each fragment ion was extracted based on the predicted m/z and a mass window around the ion, set to 2 times 60,000 resolution (FWHM) at 400 m/z.

## Electronic supplementary material

Supplementary Information

## Data Availability

Data will be made available upon request to the corresponding authors.

## References

[1] Bunnage ME, Chekler EL, Jones LH (2013). Target validation using chemical probes. Nat Chem Biol.

[2] Bunnage ME, Gilbert AM, Jones LH, Hett EC (2015). Know your target, know your molecule. Nat Chem Biol.

[3] Morgan P (2018). Impact of a five-dimensional framework on R&D productivity at AstraZeneca. Nat Rev Drug Discov.

[4] Advani RH (2013). Bruton tyrosine kinase inhibitor ibrutinib (PCI-32765) has significant activity in patients with relapsed/refractory B-cell malignancies. J Clin Oncol.

[5] Evans EK (2013). Inhibition of Btk with CC-292 provides early pharmacodynamic assessment of activity in mice and humans. J Pharmacol Exp Ther.

[6] Janes MR (2018). Targeting KRAS Mutant Cancers With a Covalent G12C-Specific Inhibitor. Cell.

[7] Gao X, Le X, Costa DB (2016). The safety and efficacy of osimertinib for the treatment of EGFR T790M mutation positive non-small-cell lung cancer. Expert Rev Anticancer Ther.

[8] Smith CI (2017). From identification of the BTK kinase to effective management of leukemia. Oncogene.

[9] Downward J (2014). RAS’s cloak of invincibility slips at last?. Cancer Cell.

[10] Lito P, Solomon M, Li LS, Hansen R, Rosen N (2016). Allele-specific inhibitors inactivate mutant KRAS G12C by a trapping mechanism. Science.

[11] Ostrem JM, Peters U, Sos ML, Wells JA, Shokat KM (2013). K-Ras(G12C) inhibitors allosterically control GTP affinity and effector interactions. Nature.

[12] Papke B, Der CJ (2017). Drugging RAS: Know the enemy. Science.

[13] Patricelli MP (2016). Selective Inhibition of Oncogenic KRAS Output with Small Molecules Targeting the Inactive State. Cancer Discov.

[14] Strelow JM (2017). A Perspective on the Kinetics of Covalent and Irreversible Inhibition. SLAS Discov.

