# Supplementary material for: An Internally Controlled Quantitative Target Occupancy Assay for Covalent Inhibitors
Source: Sci Rep. 2018 Sep 25;8:14312. doi: 10.1038/s41598-018-32683-w (PMC6156402; doi:10.1038/s41598-018-32683-w)

An Internally Controlled Quantitative Target Occupancy Assay for Covalent Inhibitors

Rasmus Hansen*, Sarah J Firdaus, Shuangwei Li, Matthew R Janes, Jingchuan Zhang, Yi Liu &
Patrick P Zarrinkar*

Supplementary Information

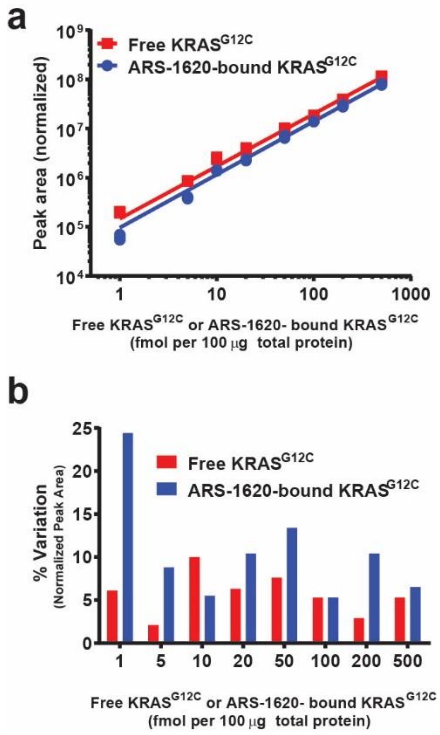

Supplementary Figure S1. Assay validation for $KRAS^{G12C}$ occupancy by ARS-1620 in human tumor lysate. The lysate was generated from a commercially obtained fresh-frozen clinical specimen. The specimen was from a resection of a $KRAS^{G12C}$-negative lung tumor, and recombinant free and ARS-1620-bound $KRAS^{G12C}$ protein were independently spiked into the lysate as indicated. (**a, b**) Confirmation of l.o.d. and l.o.q. of free and ARS-1620-bound $KRAS^{G12C}$ protein. (**a**) Sensitivity of detection. Individual replicates are shown, and for most points closely overlap. A fit of the data to straight lines yielded R^2 values of 0.99 for both free and ARS-1620-bound $KRAS^{G12C}$. (**b**) Percent variation calculated as the percent of the mean of the range of values observed [100 x ((max value – min value)/mean)] at different amounts of total spiked $KRAS^{G12C}$ protein, calculated from the replicates shown in (**a**).

Supplementary Table S1. Accuracy and precision of target occupancy measurements at different levels of KRAS^{G12C} engagement by ARS-1620 determined from recombinant free and ARS-1620-bound KRAS^{G12C} spiked into lysates from KRAS^{G12C}-negative mouse A375 xenograft tumors.

% KRAS^{G12C} engagement (True value of spiked mixture of free and ARS-1620 bound KRAS^{G12C})	Experimental KRAS^{G12C} engagement by ARS-1620			Average	% Variation*	Percentage point deviation from true value
	Technical replicate 1	Technical replicate 2	Technical replicate 3			
0	0	0	0	0.0	0.0	0
10	18	17	16	17	12	7
25	31	30	31	31	3.3	6
50	57	57	57	57	0.0	7
75	78	79	78	78	1.3	3
90	89	89	89	89	0.0	-1
100	100	100	100	100.0	0.0	0

*Percent Variation calculated as the percent of the mean of the range of values observed [100 x ((max value – min value)/mean)] for the replicates shown.

Supplementary Table S2. Accuracy and precision of target occupancy measurements at different levels of $KRAS^{G12C}$ engagement by ARS-1620 determined from recombinant free and ARS-1620-bound $KRAS^{G12C}$ spiked into lysate from a $KRAS^{G12C}$-negative human lung tumor specimen.

% $KRAS^{G12C}$ engagement (True value of spiked mixture of free and ARS-1620 bound $KRAS^{G12C}$)	Experimental $KRAS^{G12C}$ engagement by ARS-1620			Average	% Variation*	Percentage point deviation from true value
	Technical replicate 1	Technical replicate 2	Technical replicate 3			
0	0	0	0	0.0	0.0	0
10	10	11	11	11	9.1	1
25	24	25	26	25	7.8	0
50	52	53	50	52	5.8	2
75	68	71	70	70	4.3	-5
90	85	87	87	86	2.3	-4
100	100	100	100	100.0	0.0	0

*Percent Variation calculated as the percent of the mean of the range of values observed [100 x ((max value – min value)/mean)] for the replicates shown.

Supplementary Table S3. Reproducibility of $KRAS^{G12C}$ engagement measurements.

Tumor sample #	Treatment Time	% G12C engagement				% Variation*
		Experiment 1	Experiment 2	Experiment 3	Experiment 4	
1	1 h	32	29	26	33	23
2	1 h	33	26	21	33	42
3	6 h	59	57	60	66	15
4	6 h	71	65	68	75	14

Engagement was assessed by analyzing aliquots of the same tumor lysate over a four week period. Four individual tumor lysates were prepared from MIA Paca-2 tumor xenografts after mice had received a single dose of 200 mg/kg ARS-1620. *Percent Variation calculated as the percent of the mean of the range of values observed [100 x ((max value – min value)/mean)] for the replicates shown.

Supplementary Table S4. Mass spectrometry detection of free and ARS-1620-modified peptides.

Name	Peptide sequence	Precursor peptide ions (m/z)	Fragment ions
KRAS^{G12C} (residues 6 - 16) (cysteine alkylated with iodoacetamide)	LVVVGAC[+57.0]GVGK	529.8050 / 533.8121 (z=2)	$y7^{+}$, $y8^{+}$
KRAS^{G12C}- ARS-1620 (residues 6 – 16)	LVVVGAC[+430.1]GVGK	477.8989 / 480.5703 (z=3)	$y7^{2+}$, $y8^{2+}$
KRAS / NRAS normalization peptide (residues 136- 147 in KRAS and NRAS)[a]	SYGIPFIETSAK	656.8428 / 660.8499 (z=2)	$y7^{+}$, $y8^{+}$

[a]This peptide is only quantitated in the context of the experiments using the conventional vehicle controlled method shown in comparison to our new method in Fig. 1d, as described in ref. 6. The peptide is produced in the tryptic digest from both KRAS and NRAS. The sequence and position is identical in KRAS and NRAS.

Uncropped version of the gel image shown in Fig. 1a

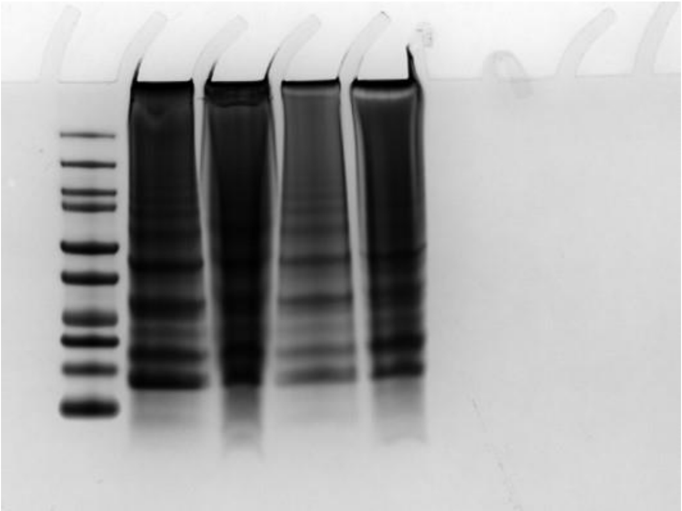

Supplement: Supplementary file 1 — Supplementary Information [file 41598_2018_32683_MOESM1_ESM.pdf]
